# COVID-19 vaccination hesitancy and willingness among pregnant women in Italy

**DOI:** 10.3389/fpubh.2022.995382

**Published:** 2022-10-03

**Authors:** Grazia Miraglia del Giudice, Lucio Folcarelli, Annalisa Napoli, Francesco Corea, Italo Francesco Angelillo, The Collaborative Working Group, Annalisa Agangi

**Affiliations:** Department of Experimental Medicine, University of Campania “Luigi Vanvitelli”, Naples, Italy

**Keywords:** pregnancy, vaccination, vaccine hesitancy, COVID-19, Italy

## Abstract

**Background:**

Pregnant women, especially those with comorbidities, compared to those non-pregnant, have higher risk of developing a severe form of COVID-19. However, COVID-19 vaccine uptake is very low among them.

**Methods:**

An anonymous questionnaire was administered to randomly selected women 18 years of age that were currently pregnant or had just given birth between September 2021 and May 2022 in the geographic area of Naples. Vaccine hesitancy was assessed using the vaccine hesitancy scale (VHS).

**Results:**

A total of 385 women participated. Women who had not been infected by SARS-CoV-2 and who needed information about vaccination against COVID-19 had a higher perceived risk of being infected with SARS-CoV-2. More than half (54.3%) of the women were very afraid of the potential side effects of the COVID-19 vaccination on the fetus. There was higher concern of the side effects of the vaccine on the fetus among those who did not have a graduate degree, those with high-risk pregnancy, those who had not been infected by SARS-CoV-2, those who were more concerned that they could be infected by SARS-CoV-2, those who did not know that this vaccination was recommended for them, and those trusting mass media/internet/social networks for information. Only 21.3% were vaccinated when pregnant, mostly women with a university degree, those who had been infected by SARS-CoV-2 before pregnancy, those who did not need information, and those who acquired information about the vaccination from gynecologists. Almost three-quarters (71.9%) were willing to receive the vaccination and those more likely were those with a university degree, those who have had at least one relative/cohabitant partner/friend who had been infected by SARS-CoV-2, those who were more concerned that they could be infected by SARS-CoV-2, and those who were not extremely concerned of the side effects of the vaccine on the fetus. A total of 86.4% were highly hesitant. Highly hesitant were respondents who did not get a graduate degree, those less concerned that they could be infected by SARS-CoV-2, and those trusting mass media/internet/social networks for information.

**Conclusion:**

Public health efforts and education campaigns for pregnant women are needed for changing their perception patterns and for supporting gynecologists in promoting the uptake of this vaccination.

## Introduction

The pandemic caused by the new strain of coronavirus (SARS-CoV-2) is still affecting more than 200 countries and by August 23, 2022, over 590 million confirmed cases of Coronavirus disease 2019 (COVID-19) and 6.45 million deaths had been reported globally (1). Public health measures in communities remain the foundation to prevent and to reduce the spread of the SARS-CoV-2 infection. It is well-known that these universal measures include hand washing with soap and water, wearing of face masks, social distancing, covering of the mouth and nose when coughing, and avoiding touching the face. Moreover, the availability of efficacious vaccines against SARS-CoV-2 and its variants has raised hope for the control of the pandemic (2).

In Italy, the COVID-19 vaccination program began in December 2020 for priority groups, including healthcare workers, long-term care residents, elderly, and essential workers and in March 2021 for all adults (3). Two m-RNA COVID-19 vaccine shots have been recommended during pregnancy in any trimester (4, 5). Although pregnant women, especially those with comorbidities, compared to non-pregnant with COVID-19, are at increased risk of hospital admission, critical care, and invasive ventilation (6, 7), yet, COVID-19 vaccine uptake is very low among this group (8–10). A few recent studies have identified a number of individual profiles who would either be hesitant to receive the vaccine or refuse it despite the severity of the disease (11–14). However, to date the hesitancy and the intention about vaccination against SARS-CoV-2 of pregnant women is scarcely reported in Italy (15, 16). Taking this into consideration, a cross-sectional survey has been conducted to evaluate primarily the uptake, the hesitancy, and the willingness regarding the vaccination against the SARS-CoV-2 in a large sample of pregnant and postpartum women in Italy. Secondarily, the predictors influencing uptake, hesitancy, and intention to be vaccinated were also examined.

## Materials and methods

### Setting and population

This work is part of a larger research project toward COVID-19 vaccination among different groups of people living in Southern Italy (17–23). This survey was conducted between September 2021 and May 2022 in two public hospitals selected by simple random sampling from the list of those with a gynecology ward in the geographic area of Naples, Southern part of Italy.

The inclusion criterion consisted of women 18 years of age that were currently pregnant (from all three trimesters of gestation) or had just given birth in the 3 days before the time of the survey. Study participants were randomly approached while waiting for their regularly scheduled clinical appointment at the Gynecology and Obstetrics outpatient clinics or while attending the maternity wards located in the two hospitals.

A minimum target sample size of 380 was estimated based on the assumption that 30% of the subjects in the population were willing to receive the vaccination against COVID-19 during the pregnancy, with a margin of error of 5%, a confidence interval of 95%, and an expected response rate of 85%.

### Procedures

This study was approved by the Ethics Committee of the Teaching Hospital of the University of Campania “Luigi Vanvitelli”. A letter with the request of collaboration and the explanation of the purpose of the survey was sent to the health directors of the selected hospitals. Experienced trained personnel not involved in the clinical care approached the participants and explained the purpose, contents, and methodology of the research, that the participation was on an anonymous and voluntary basis, that all questions were compulsory, and that they were free to quit at any time. The experienced personnel conducted a face-to-face interview in a setting that was safe for both participants and personnel or a telephone interview. All participants prior to enrollment in the study gave written or verbal informed consent. No gifts or monetary compensation was provided to participants.

### Questionnaire

The questionnaire was developed based on the content of instruments that were used in surveys conducted by some of us on the same topic enrolling different populations (17–20, 23). Piloting of the questionnaire was undertaken among 10 non-selected women to evaluate the comprehension of the questions and answers. Those involved in the pre-test were not included in the results.

The questionnaire consisted of 42 questions exploring four domains relating to the respondents: (1) socio-demographic and general characteristics, including age, marital status, education, number of children in home, whether or not they worked in healthcare, having been infected with SARS-CoV-2, and if they know someone who had been infected with SARS-CoV-2; (2) knowledge and attitude toward COVID-19 infection and vaccination with three statements regarding the concern that she could be infected with SARS-CoV-2, the vaccine recommendation for pregnant women, and the concern of potential side effects of the vaccine on the fetus; (3) COVID-19 vaccination receipt was determined and women were considered vaccinated if they reported having received ≥1 dose or fully vaccinated before or during pregnancy (independent of the term of pregnancy). If vaccination had or had not been received, the women were asked to select from predefined answers relevant to their decision or to complete an open field question. The intention to be vaccinated against COVID-19 was investigated among those unvaccinated by asking if they were willing to receive it and the reason(s) in favor or against the vaccination. This survey item was designed by using a close-ended multiple-choice question with options, in which respondents could select all that apply. Vaccine hesitancy was assessed using the 10-item Vaccine Hesitancy Scale (VHS) adapted to COVID-19 (24, 25). Each of the 10 items was assessed on a 5-point Likert scale. The wording of the VHS was slightly modified, and the questions were adapted to refer to oneself on COVID-19 vaccination during the pregnancy; and (4) sources of information related to COVID-19 vaccination in pregnancy. Options included gynecologist or other healthcare workers, family, friends, social networks, other internet sites, and mass-media, as well as, other and none. Finally, whether they would like to receive additional information.

### Statistical analysis

Descriptive statistics were used to determine the socio-demographic and the general profile of the respondents. To explore the association between each of the independent characteristics and the outcomes of interest, a chi-square test and a Student's *t*-test were carried out for the categorical and for the continuous variables, respectively. The independent characteristics with a *p* ≤ 0.25 in the bivariate analyses were incorporated into five multivariate linear and logistic regression models to address their possible role on the following dependent variables: perceived concern that she can be infected by the SARS-CoV-2 (continuous) (Model 1); concern of potential side effects of the COVID-19 vaccine on the fetus (not at all concerned, slightly concerned, uncertain, moderately concerned = 0; extremely concerned = 1) (Model 2); having received ≥1 dose of the COVID-19 vaccine during pregnancy (no = 0; yes = 1) (Model 3); willingness to receive the vaccine against COVID-19 (no = 0; yes = 1) (Model 4); and COVID-19 vaccine high hesitancy (no = 0; yes = 1) (Model 5). The following independent variables have been selected because they are potentially related to all dependent variables: age in years, marital status, baccalaureate/graduate degree, working in healthcare, at least one other child, at-risk pregnancy, at least one chronic disease, at least one relative/cohabitant partner/friend who had contracted SARS-CoV-2, and the need for additional information on COVID-19 vaccinations. The following variables were also included in the different models: having been infected by SARS-CoV-2 in Models 1, 2, 4, and 5; having been infected by SARS-CoV-2 before the pregnancy and concern that she can be infected by the SARS-CoV-2 going to the gynecologist in Model 3; perception of their health status during pregnancy in Models 1 to 3; knowing the recommendation of the COVID-19 vaccine for pregnant women in Model 2; having received the influenza vaccination in the past year in Models 3 to 5; having received the COVID-19 vaccine in Model 1; having not received the COVID-19 vaccine because they believed that it was not effective in Model 5; concern that she can be infected by the SARS-CoV-2 in Models 2, 4, and 5; belief that COVID-19 is a serious illness for the fetus if contracted during the pregnancy in Models 2 to 5; concern of the potential side effects of the COVID-19 vaccine on the fetus in Model 4; most trusted source of information related to the COVID-19 vaccination being the gynecologist in Models 3 and 4; and most trusted source of information related to the COVID-19 vaccination being mass media/internet sites/social networks in Models 1, 2, and 5. The variables with *p* = 0.2 and *p* = 0.4 were retained or excluded from the multivariate models by using a stepwise forward selection method, respectively. Results of the logistic regression models were measured using Odds Ratios (ORs) together with their 95% confidence intervals (CIs), whereas results of the linear regression models used standardized regression coefficients (ß). All analyses were based on two-sided *p*-values, with statistical significance defined as *p* ≤ 0.05. STATA statistical software version 15.1 was used to analyze the data.

## Results

### Characteristics of the respondents

A total of 406 pregnant women were approached and 385 agreed to participate in this study giving a response rate of 94.8%. The main characteristics of the study population are summarized in Table 1. The mean age was 32.2 years, the vast majority were married or were living with a partner, less than one-fourth had completed a university degree, the majority had at least one other child at home, 32.5% were in the third trimester of pregnancy, 32.5% had been infected by SARS-CoV-2 and 47.2% of which were infected during their pregnancy, 52.8% reported being previously infected by COVID-19, and 15.3% had one or more comorbidities.

**Table 1:** Socio-demographic and key characteristics of the study population.

**Characteristics**	**N**	**%**
Age, years	32.2 ± 5.4 (19–46)^*^	
Marital status		
Married/cohabited with a partner	349	90.7
Unmarried/separated/divorced/widowed	36	9.3
Educational level		
High school degree or less	293	76.1
Baccalaureate/graduate degree	92	23.9
Employment		
Worker in healthcare	12	3.1
Other	373	96.9
Number of children		
0	172	44.7
≥1	213	55.3
Trimester of pregnancy		
First	5	1.3
Second	19	4.9
Third	125	32.5
Given birth	236	61.3
Having been infected by SARS-CoV-2		
No	260	67.5
Yes	125	32.5
During pregnancy	59	47.2
Before pregnancy	66	52.8
Pregnancy at risk		
No	265	68.8
Yes	120	31.2
At least one chronic disease		
No	326	84.7
Yes	59	15.3
At least one relative/cohabitant partner/friend who had been infected by SARS-CoV-2		
No	65	16.9
Yes	320	83.1

* Mean ± Standard deviation (range).

### Attitude toward COVID-19

The overall mean value of the respondent's subjective perception of the risk of being infected by SARS-CoV-2, measured with a ten-point Likert scale ranging from 1 representing not at all to 10 representing extremely likely, was 6.7 with 26.5% that responded with a value of 10. Potential predictors of the different outcomes tested in the multivariate linear and logistic regression analysis are shown in Table 2. Women had a significantly higher level of concern of being infected by SARS-CoV-2 if they had not been infected by it and if they needed additional information about vaccination against COVID-19 (Model 1). More than half (54.3%) of the women were very afraid of the potential side effects of the vaccination against COVID-19 on the fetus. The multivariate logistic regression model showed that this concern was higher among women who did not have a graduate degree, in those whose pregnancy was at risk, in those who had not been infected by SARS-CoV-2, in those with higher perceived concern of being infected by SARS-CoV-2, in those who did not know that this vaccination was recommended for pregnant women, and in those trusting mass media, internet sites, and social networks for their information about vaccination against COVID-19 (Model 2 in Table 2).

**Table 2:** Determinants of the different outcomes of interest using linear and logistic regression analysis.

**Variable**	**Coeff**.	**SE**	***t***	***p***
**Model 1**. Perceived concern of being infected by SARS-CoV-2 F (4, 380) = 6.59, *p* < 0.0001, R^2^ = 6.5%, adjusted R^2^ = 5.5%				
Need to receive additional information about COVID-19 vaccine during pregnancy	0.98	0.32	3.05	0.002
Not having been infected by SARS-CoV-2	−0.78	0.31	−2.52	0.012
Not having been vaccinated against COVID-19	−0.44	0.31	−1.44	0.151
Older	0.02	0.02	0.85	0.395
	**OR**	**SE**	**95% CI**	***p***
**Model 2**. Extremely concerned of the potential side effects of the vaccine against COVID-19 on the fetus Log likelihood = −219.58, χ^2^ = 90.16 (9 df), *p* < 0.0001				
Trusting mass media, internet sites, and social networks for their information about the COVID-19 vaccine	2.80	0.82	1.57–4.98	< 0.001
Not knowing that the COVID-19 vaccine was recommended for pregnant women	0.31	0.09	0.17–0.54	< 0.001
Not having baccalaureate/graduate degree	0.39	0.11	0.22–0.69	0.001
Higher perceived concern of being infected by SARS-CoV-2	1.14	0.05	1.04–1.24	0.002
Not having been infected by SARS-CoV-2	0.51	0.13	0.31–0.84	0.008
Pregnancy at risk	1.87	0.51	1.09–3.19	0.022
Need to receive additional information about COVID-19 vaccine during pregnancy	1.52	0.41	0.89–2.61	0.122
Unmarried	0.53	0.22	0.23–1.22	0.139
Lower self-rated health status during pregnancy	0.94	0.06	0.83–1.07	0.392
**Model 3**. Having received ≥ 1 dose of the COVID-19 vaccine during pregnancy Log likelihood = −167.63, χ^2^ = 58.02 (9 df), *p* < 0.0001				
Having been infected by SARS-CoV-2 before pregnancy	4.33	1.39	2.31–8.12	< 0.001
Trusting gynecologists for their information about the COVID-19 vaccine	2.92	0.92	1.58–5.42	0.001
No need to receive additional information about COVID-19 vaccine during pregnancy	0.41	0.14	0.21–0.79	0.009
Having baccalaureate/graduate degree	1.92	0.61	1.03–3.57	0.038
Believing that COVID-19 is a serious disease when contracted during pregnancy	1.48	0.41	0.85–2.57	0.158
Having received the influenza vaccine over the past year	1.85	1.01	0.64–5.36	0.252
Higher self-rated health status during pregnancy	1.10	0.09	0.93–1.29	0.24
Pregnancy not at risk	0.68	0.23	0.35–1.32	0.263
**Model 4**. Willingness to receive the vaccine against COVID-19 Log likelihood = −128.33, χ^2^ = 39.15 (7 df), *p* < 0.0001				
Higher perceived concern of being infected by SARS-CoV-2	1.19	0.06	1.06–1.32	0.002
Having baccalaureate/graduate degree	5.24	3.39	1.47–18.65	0.01
Not being extremely concerned of the potential side effects of the vaccine against COVID-19 on the fetus	0.46	0.17	0.22–0.94	0.035
Having at least one relative/cohabitant partner/friend who had been infected by SARS-CoV-2	2.06	0.74	1.02–4.18	0.044
Having at least one chronic disease	2.73	1.45	0.96–7.76	0.059
Believing that COVID-19 is a serious disease when contracted during pregnancy	1.35	0.43	0.72–2.53	0.339
Need to receive additional information about COVID-19 vaccine during pregnancy	1.37	0.46	0.70–2.67	0.354
**Model 5**. COVID-19 vaccine high hesitancy during pregnancy Log likelihood = −81.87, χ^2^ = 34.77 (4 df), *p* < 0.0001				
Trusting mass media, internet sites, and social networks for their information about the COVID-19 vaccine	6.18	2.81	2.53–15.09	< 0.001
Lower perceived concern of being infected by SARS-CoV-2	0.77	0.07	0.64–0.93	0.007
Not having baccalaureate/graduate degree	0.38	0.17	0.15–0.92	0.033
Not having received the vaccine because the vaccine was not effective	2.69	2.09	0.59–12.34	0.201

### COVID-19 vaccine behavior and willingness

Of the respondents, 136 (35.3%) had received the vaccine against COVID-19 with only 82 having received the vaccine during the pregnancy for an overall prevalence of 21.3%. Of these 82 women, 32 were fully vaccinated during pregnancy, 42 received the first dose before pregnancy, and 8 received only the first dose during pregnancy. The multivariate logistic regression model performed with having had the COVID-19 vaccine during the pregnancy as an outcome variable showed that four independent predictors were significantly associated. Women with a university degree, those who have been infected by SARS-CoV-2 before the pregnancy, those who did not need additional information about vaccination against COVID-19, and those whose most trusted source of information about vaccination against COVID-19 were gynecologists were more likely to have received this vaccine (Model 3 in Table 2). The main reasons for having received the vaccination were for the protection of themselves (79.4%), of the newborn (64.7%), and of the family members (54.4%). The main reasons for those who did not receive this vaccination during pregnancy included concerns that the vaccine is not safe (58.6%), the gynecologist did not recommend it (36.9%), and a lack of knowledge (24.9%). Among those unvaccinated, almost three-quarters (71.9%) were willing to receive the vaccination. The results of the multivariate logistic regression model revealed that women with a university degree, those who have had at least one relative/cohabitant partner/friend who had been infected by SARS-CoV-2, those with higher perceived concern of being infected by SARS-CoV-2, and those who were not extremely concerned about the potential side effects of the COVID-19 vaccine on the fetus were more likely to be willing to receive the vaccine against COVID-19 (Model 4 in Table 2). Among the respondents who intend to get a COVID-19 vaccine, the main reasons given were for the protection of themselves (82.7%), of the newborn (82.1%), and of the family members (79.3%), whereas among those who did not intend to get this vaccine, leading reasons were concern about side effects (78.5%) and efficacy (37.1%), followed by thinking that it is not safe during the pregnancy (25.7%).

### COVID-19 vaccine hesitancy

Among the women who did not receive the vaccination, the vast majority (86.4%) were highly hesitant, with a VHS score ≥25. The distribution of responses for each item on the VHS is presented in Table 3. A total of 80.3% respondents either disagreed or were undecided about whether the COVID-19 vaccines are effective during pregnancy, 85.2% strongly agreed or agreed that they were concerned about serious adverse effects, and more than one-third strongly agreed or agreed that these vaccines carried more risks than older vaccines. Less than one-third strongly agreed or agreed that the COVID-19 vaccine is important for their health (27.7%) and that vaccines are a good way to protect their newborn from the disease (22.9%). Results of the final multivariate logistic regression model revealed that three factors were significantly associated with the high hesitancy toward anti-COVID-19 vaccination. Respondents who did not have a graduate degree, those who were less concerned about the risk of being infected by SARS-CoV-2, and those trusting mass media, internet sites, and social networks for their information about vaccination against COVID-19 were more likely to be highly hesitant (Model 5 in Table 2).

**Table 3:** Descriptive characteristics of respondents' VHS index about the COVID-19 vaccine.

**Item**	**Participants' response**	**N**	**%**
Getting vaccinated against COVID-19 during pregnancy is important for my health	Disagree Not sure Agree	111 69 69	44.6 27.7 27.7
Getting vaccinated against COVID-19 during pregnancy is efficacy	Disagree Not sure Agree	106 94 49	42.5 37.8 19.7
It is important to get COVID-19 vaccine during pregnancy to protect the newborn	Disagree Not sure Agree	124 68 57	49.8 27.3 22.9
Being vaccinated against COVID-19 during pregnancy is useful	Disagree Not sure Agree	122 78 49	49 31.3 19.7
The COVID-19 vaccine is more dangerous than the other vaccines administered during pregnancy (such as diphtheria, tetanus, pertussis, influenzae)	Disagree Not sure Agree	78 62 109	31.3 25 43.7
The information I receive from the Ministry of Health on the COVID-19 vaccine during pregnancy is reliable	Disagree Not sure Agree	113 76 60	45.4 30.5 24.1
Getting the COVID-19 vaccine during pregnancy is an effective strategy to protect me from the disease	Disagree Not sure Agree	124 60 65	49.8 24.1 26.1
I follow my gynecologist's advice about getting the COVID-19 vaccine during pregnancy	Disagree Not sure Agree	80 29 142	31.3 11.7 57
I am worried about a serious side effect after getting the COVID-19 vaccine during pregnancy	Disagree Not sure Agree	27 10 212	10.8 4 85.2
I do not need the COVID-19 vaccine during pregnancy	Disagree Not sure Agree	85 55 105	35.7 22.1 42.2

### Sources of COVID-19 vaccination-related information

Almost all women reported that they had received information about vaccination against COVID-19 (98.7%). In the multiple-choice question regarding the sources of information, gynecologists (61%), internet (59.2%), and mass media (54.5%) were the most trusted sources. Almost one-third of the respondents needed to receive additional information about vaccination against COVID-19 (29.3%).

## Discussion

This survey is among the first to provide an insight on the coverage, hesitancy, and willingness to receive the SARS-CoV-2 vaccination among pregnant women in Italy, as well as to identify factors that were related to an individual's decision.

A striking observation in the results of this study was the very low number of women (21.3%) that claimed that they had received at least one dose of the COVID-19 vaccine during pregnancy. A higher coverage has been observed in developed countries such as Japan with a value of 82.1% (26), Canada with 48.2% (27), New Zealand with 44% (28), whereas lower values of 20.8, 18.1, 10.5, and 1.2% have been found respectively in Israel (29), in Norway and Sweden (30), in the United Kingdom (8) and in Germany (31). Interestingly, very low uptake also of other recommended vaccines among pregnant women have been reported in the literature, including for example results from Italy with none having received tetanus, diphtheria, and acellular pertussis vaccine, and only 1.4% for influenza (32), and from Tunisia, France, United States, and Peru respectively with 4.6% (33), 7.4% (34), 10.3% (35), and 19% (36) for influenza. These findings underline the need to promote education intervention, especially during pregnancy, in order to improve women's knowledge on the benefits of antenatal recommended vaccinations. Not surprisingly, women who did not get the COVID-19 vaccine or did not intend to receive it indicated as major reasons the fears about its side effects and doubts about its efficacy. This fear of adverse events was already observed as a prevalent reason for refusing the COVID-19 vaccination in other studies in Italy (15) and elsewhere (13, 37–41). Among the unvaccinated participants, 71.9% reported their willingness to receive the vaccine. This frequency is lower compared to the values of 84.1% (20) and of 80.7% (19) observed by some of us in the same geographic area among different groups of individuals. However, the proportion was considerably higher than the values reported in several other studies among pregnant women: 13.8% in Germany (31), 16.7% in Ukraine (41), 29.5% in France (38), 29.7% in Switzerland (42), 37% in Turkey (43), 43% in the United States (11), and 60.8% in Thailand (44). Moreover, the observed finding is consistent with those found in Czechia and in China with respectively, 76.6% (45) and 77.4% (46) of pregnant populations willing to receive the vaccine. Nevertheless, it is important to underline that the differences in the access to the vaccination services, in the various periods of time the studies have been conducted, in the methodologies used, and in the characteristics of the samples may hinder comparison between studies.

It is noteworthy to mention that the results of this survey provide important insight into the main sources commonly utilized by pregnant women to obtain information on COVID-19 vaccinations. Gynecologists were identified as the main source thus pinpointing their unique opportunity for the delivery of reliable information on how to prevent the disease and about its vaccine to this population. Indeed, gynecologists are the most familiar physicians with the conditions of pregnant women and, therefore, the information and recommendations they provide can directly affect vaccination decisions. This is confirmed by the finding that the women who had received information from the gynecologist were more likely to be vaccinated. This study contributes to the ample literature showing that communication and recommendations from healthcare providers are powerful factors in addressing vaccine concerns and promoting adherence to immunization schedules (47, 48). However, a large proportion of pregnant women report seeking online sources for information, and this is of concern since vaccine-hesitant groups are very active in the media environment, and most information from this source is anti-vaccination. Such content has had a negative impact on the attitudes toward vaccines and vaccine hesitancy. Indeed, pregnant women who had acquired information on the COVID-19 vaccination from online sources were more likely to perceive that the vaccine is dangerous for the fetus and to be highly hesitant. These results corroborate the findings of previously similar studies conducted elsewhere (12, 49). Moreover, it is disturbing the finding that 23.9% of the sample did not get the COVID-19 vaccine because the gynecologist did not recommend it. Therefore, there is need for further education for the gynecologists on existing guidelines to increase vaccination rates. The need of additional information also has a significant impact. Indeed, pregnant women who would like to get additional information were more likely to be concerned about being infected by SARS-CoV-2, whereas those who did not have this need were more likely to be vaccinated. These associations underlined the importance of the information on immunization in improving the level of knowledge and in changing intentions toward vaccination. The findings have been acknowledged among different populations in several geographic areas (17–19, 23, 50, 51).

The results of the present survey on the factors affecting the different outcomes of interest showed several additional significant associations. Among the socio-demographic characteristics, the level of education was the only significant factor associated with several outcomes. Indeed, pregnant women with a university degree were more likely to be vaccinated and to be willing to receive the vaccination, whereas those without a degree were more likely to be vaccine highly hesitant. These interesting findings highlight the positive impact of education on the vaccine uptake and on the attitudes toward vaccination as also previously found in the literature (39, 42). Moreover, the current study discovered that the evidence of a personal prior infection with SARS-CoV-2 or in a relative/cohabitant partner/friend was linked with several outcomes. Women who have had a prior infection were more likely to have been vaccinated and those who have had such experience in a relative/cohabitant partner/friend were more likely to be willing to receive the vaccination. These findings may be explained by the fact that these women may have had health consequences or have been well-informed about the negative effects of this infection and, therefore, see the vaccination as a positive intervention, whereas those without such experience may be less informed about the consequences. Finally, as expected, women with a lower perceived concern of being infected by SARS-CoV-2 were more likely to be vaccine highly hesitant. This finding underlined the need for educational campaigns and appropriate communication on this group also, because, as already reported, respondents who were vaccine highly hesitant and were worried of the potential side effects of the vaccination on the fetus were those who had acquired information also from the internet.

The potential methodological limitations of this survey should be considered in interpreting the findings. First, this survey was conducted using a cross-sectional design and, therefore, this prevents drawing any conclusion about causality in the associations found between predictors and outcomes of interest. Second, findings of the survey may not be totally generalizable to the Italian population of pregnant women, as it has been conducted in only one geographic area. Third, participants may have answered in a socially desirable manner mainly regarding a positive attitude toward the vaccination. However, participants were assured of complete anonymity in the responses at the beginning of the interview and this may have reduced the influence of desirability bias. As such, the findings are likely to be authentic. Despite these limitations, the survey outlines useful data for policymakers and healthcare workers on this sensitive topic.

In summary, the present survey has generated solid data regarding COVID-19 vaccination uptake, hesitancy, and intention to be vaccinated of pregnant women. The findings clearly indicate a low vaccine uptake and identified a high hesitancy and unwillingness to accept this vaccination irrespective of the pandemic spread of the SARS-CoV-2 that has determined an extraordinarily high number of cases and deaths. Safety of the vaccine and the lack of recommendation by the gynecologist have been identified as the major reasons for those who did not receive this vaccine unless its safety has been widely disseminated together with the recommendation for pregnant women by the scientific and health authorities. Public health efforts and education campaigns regarding the importance of this vaccine during pregnancy are needed for changing their perception patterns and for supporting gynecologists in promoting the uptake of vaccination against COVID-19.

## Data availability statement

The raw data supporting the conclusions of this article will be made available by the authors, without undue reservation.

## Ethics statement

The study involving human participants was reviewed and approved by Ethics Committee of the Teaching Hospital of the University of Campania Luigi Vanvitelli. The patients/participants provided their written or oral informed consent to participate in this study.

## The collaborative working group

Annalisa Agangi and Antonio Sciambra (Health Direction, Evangelico Betania Hospital, Naples, Italy), Glenda Scognamiglio and Walter Longanella (Health Direction, San Paolo Hospital, Naples, Italy).

## Author contributions

GMdG, FC, and LF participated in the conception and design of the study, contributed to the data collection, data analysis, and interpretation. AN contributed to the data collection, data analysis, and interpretation. IFA designed the study, was responsible for the statistical analysis and interpretation, drafted and wrote the article. All authors have read and approved the final version of the article and agree to be accountable for all aspects of the work.

## Conflict of interest

The authors declare that the research was conducted in the absence of any commercial or financial relationships that could be construed as a potential conflict of interest.

## Publisher's note

All claims expressed in this article are solely those of the authors and do not necessarily represent those of their affiliated organizations, or those of the publisher, the editors and the reviewers. Any product that may be evaluated in this article, or claim that may be made by its manufacturer, is not guaranteed or endorsed by the publisher.

## Author disclaimer

Preliminary results have been presented at the 3rd Edition of the “Giornate della ricerca scientifica e delle esperienze professionali dei giovani” of the Italian Public Health Association (SItI), March 25-26, 2022.
